# At‐line multi‐angle light scattering detector for faster process development in enveloped virus‐like particle purification

**DOI:** 10.1002/jssc.201900441

**Published:** 2019-06-19

**Authors:** Patricia Pereira Aguilar, Irene González‐Domínguez, Tobias Amadeus Schneider, Francesc Gòdia, Laura Cervera, Alois Jungbauer

**Affiliations:** ^1^ Department of Biotechnology University of Natural Resources and Life Sciences Vienna Austria; ^2^ Department d'Enginyeria Química Biològica i Ambiental Universitat Autònoma de Barcelona Bellaterra Barcelona Spain; ^3^ Austrian Centre of Industrial Biotechnology Vienna Austria

**Keywords:** enveloped bionanoparticles, fluorescent virus‐like particles, monoliths, nanoparticle tracking analysis

## Abstract

At‐line static light scattering and fluorescence monitoring allows direct in‐process tracking of fluorescent virus‐like particles. We have demonstrated this by coupling at‐line multi‐angle light scattering and fluorescence detectors to the downstream processing of enveloped virus‐like particles. Since light scattering intensity is directly proportional to particle concentration, our strategy allowed a swift identification of product containing fractions and rapid process development. Virus‐like particles containing the Human Immunodeficiency Virus‐1 Gag protein fused to the Green Fluorescence protein were produced in Human Embryonic Kidney 293 cells by transient transfection. A single‐column anion‐exchange chromatography method was used for direct capture and purification. The majority of host‐cell protein impurities passed through the column without binding. Virus‐like particles bound to the column were eluted by linear or step salt gradients. Particles recovered in the step gradient purification were characterized by nanoparticle tracking analysis, size exclusion chromatography coupled to multi‐angle light scattering and fluorescence detectors and transmission electron microscopy. A total recovery of 66% for the fluorescent particles was obtained with a 50% yield in the main product peak. Virus‐like particles were concentrated 17‐fold to final a concentration of 4.45 × 10^10^ particles/mL. Simple buffers and operation make this process suitable for large scale purposes.

**Article Related Abbreviations**
CV: column volume
DSP: downstream processing
FL: fluorescence
Gag: group specific antigen
GFP: green fluorescence protein
HEK 293: Human Embryonic Kidney 293 cells
HIV‐1: Human Immunodeficiency Virus 1
LS: light scattering
MALS: multi‐angle light scattering
NTA: nanoparticle tracking analysis
PEI: polyethilenimine
SEC‐MALS‐FL: SEC coupled to MALS and fluorescence detectors
VLP: virus‐like particle

## 1 INTRODUCTION

The majority of the analytical techniques used in virus‐like particle (VLP) downstream processing (DSP) were imported from protein DSP 1. Typically, colorimetric methods such as Bradford assay and SDS‐PAGE are used to gain insights into the total protein content. In addition, assays such as Western blot and ELISA allow the specific detection and quantification of VLP specific proteins. However, none of these methods confirms the presence of correctly assembled VLPs, but only the presence of specific viral proteins, which, when in their free state, represent one of the product related impurities. Particle morphology is usually confirmed by high‐resolution microscopy techniques such as TEM and multi‐frequency atomic force microscopy 2. Particle number and size distributions are frequently measured by nanoparticle tracking analysis (NTA) and dynamic or static light scattering 3, 4, 5. However, these methods are non‐specific regarding the particle's composition and consider all structures with the same hydrodynamic or geometric radius as equals. As a result, a combination of biochemical, biophysical, and biological analytical methods is required to ensure an accurate detection and quantification of VLPs. This results in very laborious and time‐consuming process analytics and hinders DSP process development, which is strongly dependent on the ability to detect, quantify and characterize the product of interest as well as on the capacity to discriminate the product from its related impurities.

Multi‐angle light scattering (MALS) is one of the most used techniques for quantification and characterization of different nanoparticles 6. Usually, monodisperse samples are required to allow for structural characterization based on light scattering. Typically, SEC or asymmetric flow field‐flow fractionation are used to first separate the samples before the measurement 4, 7. Nevertheless, the intensity of scattered light by particles in solution is directly proportional to the particle concentration 8, 9. Thus, an at‐line MALS detector can be used for fast detection and semi‐quantification of particles without the requirement of monodisperse samples. This rapid method could be used as an in‐process control to speed up process development and optimization.

Outbreaks of Flu, Ebola, and Zika in the last decade reinforced the need for faster process development and flexible manufacturing platforms which enable the production of millions of vaccine doses in a short time 10, 11. Among different candidates, retroviral‐based VLPs are promising towards the development of vaccines and drug delivery systems 12, 13. Enveloped virus‐like particles (eVLPs) are produced by recombinantly expressing one or more viral proteins 10, 11, 12. As a model, we used eVLPs produced in Human Embryonic Kidney (HEK) 293 cells by recombinantly expressing the Human Immunodeficiency Virus‐1 (HIV‐1) group specific antigen (Gag) protein which was fused to Green Fluorescence protein (GFP) 2, 14, 15, 16. Similar to the native HIV‐1 production process, upon recombinant expression, Gag polyprotein self‐assembles underneath the cell membrane and VLPs bud to the extracellular space as spherical particles 17. The resulting particles are enveloped by a host‐cell derived lipid bilayer and have a diameter of 100–200 nm 4, 18, 19. The integration of GFP permits the use of fluorescence‐based techniques as an orthogonal method for detection and quantification of HIV‐1 Gag‐GFP VLPs.

Different DSP strategies have been developed for the purification of bionanoparticles 5, 11, 20, 21, 22. Monolithic columns have been often used for the direct capture and purification of enveloped virus and VLPs from cell culture supernatant 23, 24, 25, 26, 27. Due to its convective pore structure, high binding capacities can be achieved in monolithic columns while maintaining high flow rates. This results in higher productivities when compared with traditional VLP purification methods such as density gradient centrifugation 28. As a model purification strategy, we used anion exchange monolithic columns for the direct capture and purification of HIV‐1 Gag‐GFP VLPs directly from cell culture supernatant. We show how at‐line MALS and fluorescence detectors simplified DSP process development and optimization. Furthermore, size exclusion chromatography coupled to multi‐angle light scattering and fluorescence (SEC‐MALS‐FL) is an effective method for particle quantification and characterization.

## 2 MATERIALS AND METHODS

### 2.1 Chemicals

The chemicals used for all experiments were acquired from Merck (Darmstadt, Germany) or Sigma Aldrich (St. Louis, MO, USA).

### 2.2 Production of virus‐like particles

#### 2.2.1 Cell line, media, and culture conditions

A serum‐free suspension‐adapted Human Embryonic Kidney 293 cells (HEK 293) cell line (HEK293SF‐3F6, National Research Council, Montreal, Canada) kindly provided by Dr. Amine Kamen from McGill University (McGill, Montreal, Canada) was used. Cells were cultured in Freestyle 293® medium supplemented with 0.1% Pluronic® (both Invitrogen, Carlsbad, CA, USA), 1.6 mg/L of r‐transferrin (Merck Millipore, Kankakee, IL, USA), 19.8 mg/L of r‐insulin (Novo Nordisk Pharmatek, Køge, Denmark), and 0.9X of an in‐house developed lipid mixture to maximize cell growth 8. Cells were routinely maintained in 20 mL of culture medium. Flasks were shaken at 130 rpm using an orbital shaker (Stuart, Stone, UK) placed in an incubator maintained at 37˚C in a humidified atmosphere of 5% CO_2_ in air.

#### 2.2.2 Plasmids

The pGag‐eGFP plasmid used in this work codes for a Rev‐independent HIV‐1 Gag protein fused in frame to the enhanced GFP 27. The plasmid from the NIH AIDS Reagent Program (Cat 11468) was constructed by cloning the Gag sequence from pCMV55M1‐10 28 into the pEGFP‐N1 plasmid (Clontech, Takara Bio, Mountain View, CA, USA). The plasmids were prepared and purified as previously described with Endofree Plasmid Mega kit (Qiagen, Hilden, Germany) 29. Snap Gene Viewer was used to analyse the plasmid features (GSL Biotech, Chicago, IL, USA).

#### 2.2.3 DNA/polyethilenimine complex formation and transient transfection of HEK 293 cells

HEK 293 suspension cells were transiently transfected using 25 kDa linear polyethilenimine (PEI) (PolySciences, Warrington, FO, USA). Transfections were performed using a final DNA concentration of 1 µg/mL. PEI/DNA complexes were formed by adding PEI to plasmid DNA (1:2 w/w DNA:PEI ratio) diluted in fresh culture medium (10% of the total culture volume to be transfected) 8.

Cells were cultured for 72 h post transfection to maximize VLP yields 30. Cell culture supernatants were primary harvested by centrifugation at 4000 g for 30 min at 4˚C. Recovered supernatants were stored at 4˚C before purification.

### 2.3 Chromatographic purification

#### 2.3.1 Chromatographic equipment and mobile phases

Chromatographic experiments were performed using an Äkta pure 25 M2 with a sample pump S9 and fraction collector F9‐C (GE Healthcare, Uppsala, Sweden). During the purification runs, pH, conductivity and UV absorbance at 280 and 260 nm wavelengths were monitored. Unicorn software versions 5.10 or 6.4.1 (GE Healthcare, Uppsala, Sweden) were used for method programming, system control, and data acquisition.

Mobile phase A and B consisted in 50 mM HEPES, pH 7.2 and 50 mM HEPES, 2 M NaCl, pH 7.2 respectively. Sanitization buffer consisted in 1 M NaOH.

#### 2.3.2 Preparative scale purification

Clarified cell culture supernatant containing HIV‐1 Gag‐GFP VLPs was 0.8 µm filtered (Millex AA syringe filter, Millipore Bedford, MA, USA) and 100 mL were loaded into a 1 mL radial flow monolith (CIMmultus™ QA, BIA Separations, Ajdovščina, Slovenia). Before loading, the column was equilibrated with 50 mM HEPES, 100 mM NaCl, pH 7.2 (5% buffer B). After the loading phase, the column was washed with equilibration buffer (5% buffer B) for 15 column volumes (CV). In the linear gradient purification, a salt linear gradient from 100 to 1000 mM NaCl (5 to 50% buffer B) in 50 CV was used. For the step gradient purification, three steps of 300, 520 and 1000 mM NaCl (15, 26 and 50% buffer B) with 15 CV each were used for elution. In both purification strategies, the column was regenerated with 100% buffer B in a 10 CV step. After regeneration, the column was sanitized using 10 CV of 1 M NaOH. All preparative purification runs were performed using a flow rate of 1 mL/min. The sample was loaded into the column using the sample pump. Fractions of 1 mL were collected in 96 deep‐well plates and pooled according to the chromatograms.

### 2.4 Nanoparticle tracking analysis

Particle concentration and particle size distribution were determined by NTA using a NanoSight NS300 (Malvern Instruments, Worcestershire, UK) equipped with a blue laser module (488 nm), a neutral density filter and a 500 nm fluorescence filter. To obtain a particle concentration of 20 to 80 particles per video frame in the measuring chamber, samples were serially diluted using particle‐free water or 0.1 µm filtered 50 mM HEPES pH 7.2 buffer. Each sample was measured with both scattering (LS) and fluorescence (FL) modes in three different dilutions in triplicates. In total, nine videos of 60 s were recorded per sample. The camera level varied between 14 and 16 and it was manually adjusted prior to each measurement. Recorded videos were analysed using the NanoSight NTA software version 3.2 (Malvern Instruments, Worcestershire, UK). Detection thresholds between 3 and 5 were used.

### 2.5 Total protein and double stranded DNA quantification

Total protein concentration was determined by Bradford assay using Coomassie blue G‐250‐based protein dye reagent (Bio‐Rad Laboratories, Hercules, CA, USA). The calibration curve was obtained using BSA standards (Thermo Fisher Scientific, Waltham, MA, USA) diluted in TE‐Buffer to a concentration range of 50–200 µg/mL. Double stranded DNA (dsDNA) quantification was performed using the Quant‐iTTM PicoGreen® dsDNA kit (Life Technologies, Waltham, MA, USA). Protein and dsDNA assays were performed according to the respective instructions from the manufacturer in a 96‐well plate format. Since HIV‐1 Gag‐GFP VLPs emit at the same range as the Quant‐iTTM PicoGreen® reagent, the native fluorescence was measured prior to the reagent addition and later subtracted to the fluorescence after the reaction.

### 2.6 Sodium dodecyl sulfate‐polyacrylamide gel electrophoresis and Western blot analysis

Precast NuPAGE Bis/Tris gels 4–12% (Invitrogen, Carlsbad, CA, USA) were used in a MES‐SDS buffer system. The protocol was adapted from manufacturer's instructions. Briefly: 40 µL of sample were mixed with 20 µL of 4x LDS buffer and 2 M DTT to a final concentration of 1% v/v. Each sample incubated at 95°C for 20 min. SeeBlue® Plus2 Pre‐stained Protein Standard (Invitrogen, Carlsbad, CA, USA) was used as protein marker. Gels were run at 200 V, 400 mA. Coomassie Brilliant Blue G‐250 based EZBlue™ Gel Staining Reagent (Sigma Aldrich, St. Louis, MO, USA) was used for protein staining. After SDS‐PAGE, proteins were blotted using Trans‐Blot® turbo system (Bio‐Rad Laboratories, Hercules, CA, USA) with 0.2 µm nitrocellulose membranes and blocked with 3% BSA in PBS with 0.1% w/v Tween‐20 overnight. Detection of HIV‐1 Gag‐GFP protein was performed by incubation with primary mouse monoclonal antibody against HIV‐1 p24 (Icosagen AS, Tartumaa, Estonia), diluted 1:1000 in PBS‐T containing 1% BSA for 2 h. Anti‐mouse IgG conjugated with alkaline phosphatase (Sigma Aldrich, St. Louis, MO, USA), diluted 1:1000 in PBS‐T with 1% w/v BSA was used as secondary antibody. Premixed BCIP®/NBT solution (Sigma Aldrich, St. Louis, MO, USA) was used as substrate solution.

### 2.7 At‐line multi‐angle light scattering and fluorescence

At‐line MALS and fluorescence measurements were performed using an Ultimate 3000 system (Thermo Fisher, Waltham, MA, USA). A sample of all collected fractions during the purification runs was directly injected into the detectors bypassing the column. The HPLC system was equipped with a LPG‐3400SD quaternary pump, WPS‐3000TSL analytical autosampler, DAD 3000 UV‐detector and FLD 3100 fluorescence detector (Thermo Fisher, Waltham, MA, USA). Additionally, the system was connected to a multi‐angle light scattering detector DAWN HELEOS 18‐angle and a differential refractive index detector Optilab rEX (both Wyatt, Santa Barbara, CA, USA). Chromeleon 7 (Thermo Fisher Scientific, Waltham, MA, USA) and Astra 5.3.4 Wyatt, (Santa Barbara, CA, USA) software were used for method programming, system control and data acquisition. GFP fluorescence was monitored with an excitation wavelength of 480 nm and emission of 505 nm. Analysis time was 3 min/sample. Light scattering intensity was accessed by calculating the peak area of the light scattering signal obtained with the 90° angle.

### 2.8 Size exclusion chromatography coupled to multi‐angle light scattering and fluorescence

SEC‐MALS‐FL measurements were performed using the same HPLC system, detectors and software as described in Section 2.7.

A TSKgel G5000PWXL 300.0 mm × 7.8 mm i.d. in combination with a TSKgel PWXL guard column 40.0 mm × 6.0 mm i.d. (both Tosoh Bioscience, Stuttgart, Germany) were used for size exclusion chromatography. The method was previously described by Steppert et al. 20. Data analysis was done in Astra 6.1.2 using the number density procedure and the sphere model fit with a particle refractive index of 1.46 29.

### 2.9 TEM

HIV‐1 Gag‐GFP VLP samples were prepared by air‐dried negative staining method. Briefly, 8 µL of sample was placed on discharged carbon‐coated copper or holly carbon 200 mesh grids and incubated at room temperature for 1 min. Excess sample was drained carefully off the grid with filter paper. Samples were stained negatively with 8 µL of uranyl acetate (2%) by incubation for 1 min at room temperature. Excess stain was drained off as before, and grids were dried. Micrographs were taken with a JEM‐400 transmission electron microscope (JEOL USA, Pleasanton, CA, USA) equipped with an ES1000W Erlangshen charge‐coupled device camera (Model No. 785; Gatan, Pleasanton, CA, USA).

## 3 RESULTS AND DISCUSSION

In this work, we aimed to streamline the process development for enveloped virus‐like particle purification by including at‐line multi‐angle light scattering and fluorescence detectors for high‐throughput particle detection and semi‐quantification. Since it has been shown that strong anion‐exchange monoliths allow the simultaneous capture and purification of enveloped bionanoparticles such as eVLPs and exosomes 22, 23, we used a QA monolith to capture and purify HIV‐1 Gag‐GFP VLPs directly from HEK 293 cell culture supernatant in a single step. As a starting point, a salt linear gradient was used for VLP elution. Later, the data obtained in the at‐line MALS and fluorescence measurements were used to develop a salt step elution strategy, providing a base for potential scale‐up.

### 3.1 Linear gradient purification

**Figure 1:**
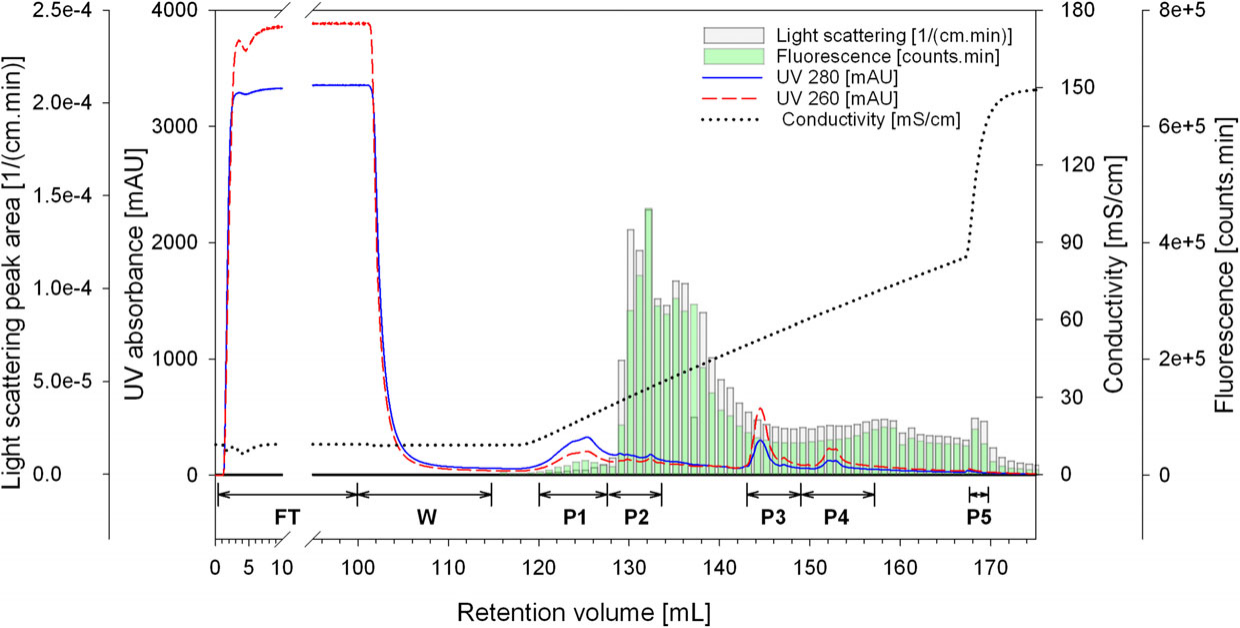
Chromatogram of the linear gradient purification of HIV‐1 Gag‐GFP VLP using a QA monolith. The loading material was 100 mL of clarified and 0.8 µm filtered HEK 293 cell culture supernatant. Bars represent the area under the curve of the light scattering intensity (grey) and fluorescence (green) at‐line measurements. FT: flow‐through; W: wash; P1‐P5: polled fractions for peaks 1–5

HIV‐1 Gag‐GFP VLPs were produced by transient transfection in HEK 293 cells. For VLP capture and purification in a single step, 100 mL of clarified and filtered cell culture supernatant were loaded into a 1 mL QA monolith. Elution was achieved by a 50 CV salt linear gradient from 100 to 1000 mM NaCl. Fractions of 1 mL were collected in 96 deep‐well plates and directly injected into the at‐line MALS and fluorescence detectors. For each elution fraction, the total light scattering intensity and the total fluorescence were calculated by integrating the signals measured by the MALS and fluorescence detectors, respectively. Data were plotted together with the purification run chromatogram (Figure 1). Since light scattering intensity is directly proportional to particle concentration 8 and the main structural element of the VLPs (Gag‐GFP protein) is fluorescent 30, this method allows a fast detection and semi‐quantification of HIV‐1 Gag‐GFP VLPs and subsequently a fast identification of the fractions containing the product of interest is possible. Considering the total light scattering intensity and the total fluorescence data, the majority of VLPs eluted from approximately 130 to 142 mL, corresponding to a conductivity range of 27–49 mS/cm. It is important to note that neither UV 280 nor UV 260 signals provide a good representation of the VLPs elution profile. This fact is one of the challenges in bionanoparticles process development, especially in early stage development where titers are usually low.

**Figure 2:**
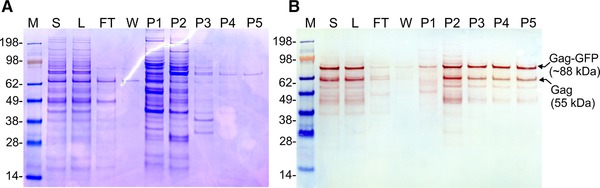
(A) SDS‐PAGE and (B) Western blot analysis of the pooled fractions from the linear gradient purification (Figure 1). M: molecular weight marker; S: cell culture supernatant; L: loading material; FT: flow‐through; W: wash; P1‐P5: pooled fractions for peaks 1–5

In order to determine the total protein and dsDNA composition of the fractions, samples were pooled according to the chromatogram (Figure 1) and analysed by Bradford and Picogreen assays respectively (Table 1). Due to the relatively high conductivity of the cell culture supernatant (11.8 mS/cm), 43% of the protein impurities did not bind to the column and were collected during column loading in the flow‐through fraction (FT). Additionally, weakly bound proteins eluted immediately at the beginning of the salt linear gradient (fraction P1). Comparable results were obtained when using conditioned media harvested prior to transfection as loading material (data not shown). Contrariwise, the majority of the dsDNA bound to the column and only 3% was recovered in the FT fraction. Elution of dsDNA was achieved at conductivities higher than 50 mS/cm and collected in the fractions P3 and P4, in which the total protein content was very low (Table 1 and Figure 2A). Western blot analysis detecting HIV‐1 p24 confirmed the presence of the Gag‐GFP protein in all elution fractions (band at approximately 88 kDa, Figure 2B, P1‐P5). Little or no signal was obtained for the fractions FT and W in the Western blot, confirming the successful binding of the VLPs to the column. According to the light scattering and fluorescence data, the fraction P2 contains the majority of the HIV‐1 Gag‐GFP VLPs. Comparing the total protein and dsDNA content of P2 with the loading material (L), a depletion of approximately 96% of total protein and 99% of dsDNA was achieved. In P3 and P4, VLPs and host cell dsDNA co‐eluted. Enveloped VLPs and dsDNA co‐elution during purification using anion‐exchange chromatography has been previously reported 23.

**Table 1:** Total protein and dsDNA mass balance of the purification of HIV‐1 Gag‐GFP VLPs using a linear gradient elution (Figure 1). S: supernatant; L: loading material; FT: flow‐through; W: wash; P1‐P5: peaks 1‐5

Sample	Volume [mL]	Total protein [µg/mL]	Total protein %	dsDNA [ng/mL]	dsDNA %
S	100	310.9	–	1387.5	–
L	100	313.1	100.0	1289.4	100.0
FT	100	134.9	43.1	37.5	2.9
W	15	<LLOQ	–	<LLOQ	–
P1	7	367.6	8.2	10.2	0.1
P2	5	275.1	4.4	310.5	1.2
P3	6	<LLOQ	–	5114.5	23.8
P4	6	<LLOQ	–	3129.6	14.6
P5	2	<LLOQ	–	1108.3	1.7
Recovery			55.7		44.3

<LLOQ: lower than the lower LOQ

The at‐line MALS and fluorescence monitoring allowed a fast identification of the VLP containing fractions and a direct transfer from the linear gradient to a step gradient elution. In the next chapter, we describe the step gradient purification.

### 3.2 Step gradient purification

**Figure 3:**
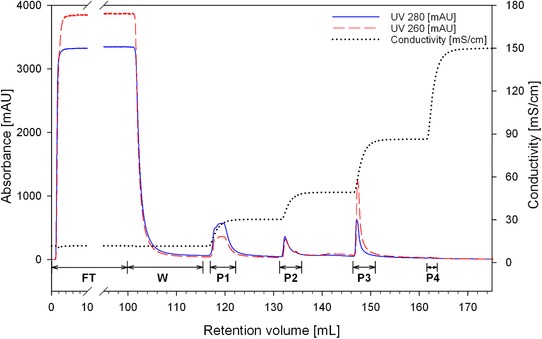
Chromatogram of the step gradient purification of HIV‐1 Gag‐GFP VLP using a QA monolith. The loading material was 100 mL of clarified and 0.8 µm filtered HEK 293 cell culture supernatant. FT: flow‐through; W: wash; P1‐P4: pooled fractions for peaks 1–4

**Figure 4:**
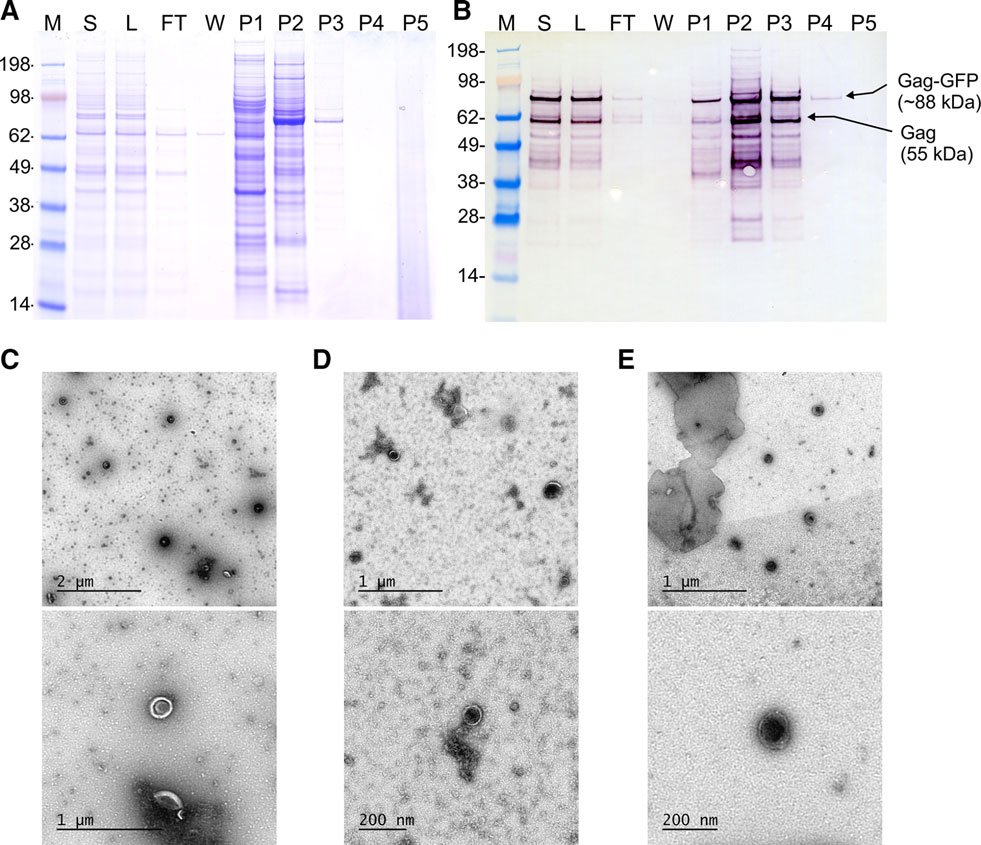
A: SDS‐PAGE and B: Western blot analysis of the pooled fractions from the step gradient purification (Figure 3). (C), (D), and (E) electron microscopy micrographs of loading material (L) and fractions P2 and P3, respectively. M: molecular weight marker; S: cell culture supernatant; L: loading material; FT: flow‐through; W: wash; P1‐P4: pooled fractions for peaks 1–4

The step gradient purification was designed based on the results obtained by the at‐line MALS and fluorescence monitoring of the linear gradient purification. The same column and loading material were used. Column equilibration, loading and regeneration conditions were also kept constant. Elution was designed targeting the recovery of the VLPs eluted in the linear gradient purification in a conductivity range of 27–49 mS/cm, as well as targeting the separation of VLPs from weakly bound protein impurities and from strongly bound dsDNA. Therefore, elution consisted in three steps of 15 CV each, using 300, 520, and 1000 mM NaCl (15, 26 and 50% B), corresponding to approximately 30, 49, and 86 mS/cm. In order to characterize the fractions collected in each step, samples were pooled according to the chromatogram (Figure 3). Protein content was analyzed by SDS‐PAGE and Western blot and total protein was quantified by Bradford analysis (Figures 4A and B, Table 2). Picogreen assay was used to determine the dsDNA content (Table 2). Particles were visualized by transmission electron microscopy (Figures 4C–E) and quantified by NTA in scattering and fluorescence modes (Table 2).

**Table 2:** Particles (diameter: 100–200 nm), total protein and dsDNA mass balance of the purification of HIV‐1 Gag‐GFP VLPs using a step gradient elution (Figure 3). L: loading material; FT: flow‐through; W: wash; P1‐P4: peaks 1‐4; CIP: cleaning‐in‐place

Sample	Volume [mL]	Particles (LS)^a^ d: 100‐200 nm [particles/mL]	Particles (LS)^a^ d: 100‐200 nm %	Particles (FL)^b^ d: 100‐200 nm [particles/mL]	Particles (FL)^b^ d: 100‐200 nm %	Total Protein [µg/mL]	Total Protein %	dsDNA [ng/mL]	dsDNA %
									
L	100	2.4 × 10^10^	100.0	2.7 × 10^09^	100.0	297.8	100.0	448.0	100.0
FT	100	<LLOQ	–	<LLOQ	–	126.7	42.5	39.2	8.8
W	15	<LLOQ	–	<LLOQ	–	41.0	2.1	645.5	21.6
P1	5	2.2 × 10^10^	4.5	1.5 × 10^09^	2.8	632.2	10.6	91.5	1.0
P2	3	1.3 × 10^11^	16.5	4.5 × 10^10^	49.9	510.3	5.1	975.4	6.5
P3	3	2.7 × 10^10^	3.3	1.2 × 10^10^	13.6	62.4	0.6	12747.8	85.4
P4	1	5.0 × 10^09^	0.2	<LLOQ	–	<LLOQ	–	691.6	1.5
CIP	15	n.d.	–	n.d.	–	456.8	23.0	<LLOQ	–
Recovery			24.5		66.3		83.9		124.8

a Particles measured in light scattering (LS) mode

b Particles measured in fluorescence (FL) mode

<LLOQ: lower than the lower LOQ

n.d.: not determined

As in the linear gradient purification, most of the protein impurities (55%) did not bind to the column or were eluted in fraction P1 using 300 mM NaCl (30 mS/cm). Western blot analysis confirmed the presence of Gag‐GFP protein in fraction P1, however only a small number of particles were recovered in this fraction (3–5% measures by NTA in scattering or fluorescence mode). This indicates the presence of fragmented particles or free Gag‐GFP protein, which did not form a correctly assembled VLP. No particles were found in the flow‐through (FT) and wash (W) fractions, confirming the efficient capture of the VLPs by the monolithic column. As expected, most particles were recovered and concentrated in fraction P2 (17–50%) and Western blot analysis confirmed the presence of the Gag‐GFP protein. TEM micrographs confirmed the presence of correctly assembled spherical particles. Comparing the total protein and dsDNA content of P2 with the loading material (L), a depletion of approximately 95% of total protein and 94% of dsDNA was achieved for the main product fraction during the step gradient purification. Lastly, as in the linear gradient purification, the majority of dsDNA (85%) was eluted using higher ionic strength (1000 mM NaCl) and collected in fraction P3 together with strongly bound particles (3–14%).

**Figure 5:**
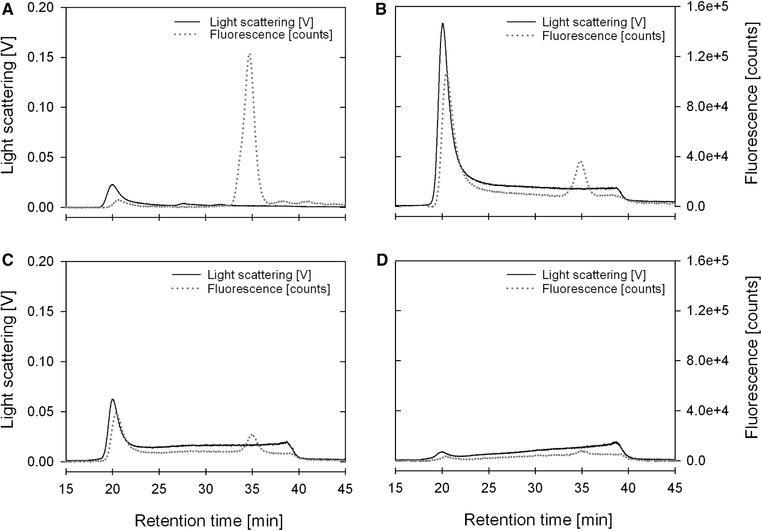
Analysis of the fractions P1‐P4 from the step gradient purification (Figure 3) by analytical size exclusion chromatography coupled to MALS and fluorescence detectors (SEC‐MALS‐FL). (A) P1; (B) P2; (C) P3; (D) P4

In order to further characterize the samples, size exclusion chromatography coupled to MALS and fluorescence detectors (SEC‐MALS‐FL) was used. This strategy allows the separation of correctly assembled HIV‐1 Gag‐GFP VLPs from free Gag‐GFP proteins and/or protein aggregates smaller than 100 nm, which are still identified when using Western blot analysis as detection method. As VLP reference, HIV‐1 gag VLPs produced in CHO cells and purified as described by Steppert et al 23 were used. As free protein reference, GFP standard was used. VLPs standard eluted in the void volume of the SEC column (approximately 20 min) while GFP standard elutes at approximately 35 min post injection (data not shown). Fractions P1, P2, P3, and P4 were analysed by SEC‐MALS‐FL (Figures 5A–D respectively). As expected, in fraction P1 (Figure 5A) a very small light scattering peak was measured at the void volume due to the very low number of particles in this fraction. On the other hand, a significant fluorescence signal was obtained at 35 min, confirming the presence of free Gag‐GFP protein already indicated by the Western blot analysis (Figure 3B). Conversely, in fraction P2 (Figure 5B) a significant light scattering signal together with a significant fluorescence signal were observed at the void volume confirming the presence of correctly assembled HIV‐1 Gag‐GFP VLPs. The small fluorescence peak observed at 35 min indicates that the sample still contains a small amount of product related impurities (free Gag‐GFP). Similarly to fraction P2, fraction P3 SEC‐MALS‐FL analysis (Figure 5C) indicates the presence of correctly assembled particles, however in lower concentration due to the lower light scattering signal intensity. This could also be observed in the measurements done by NTA (Table 2).

MALS data was also used to calculate the geometric radius of particles in fractions P2 and P3, using the Rayleigh‐Gans‐Debye approximation. Geometric radii of approximately 70 nm (140 nm of diameter) and 69 nm (138 nm of diameter) were obtained for fractions P2 and P3, respectively. These values are in agreement with the values obtained for the hydrodynamic radii of 126 and 140 nm for fractions P2 and P3, respectively, calculated using the Stokes‐Einstein equation and the diffusion constant measured by NTA.

SEC‐MALS‐FL validated at‐line MALS results as a robust analytical tool to DSP development. Furthermore, VLP integrity was confirmed by traditional TEM micrographs, where VLPs were clearly distinguished from product‐related impurities.

## 4 CONCLUDING REMARKS

The use of at‐line multi‐angle light scattering and fluorescence monitoring of a purification strategy for fluorescent VLPs allowed not only in‐process monitoring and control but also faster process development. Product‐containing fractions were quickly identified, allowing a swift transition from a linear gradient to a step gradient elution, providing a base for potential scale‐up.

The single‐step purification using a QA‐monolith effectively captured and purified HIV‐1 Gag‐GFP VLPs produced in HEK 293 cells directly from the clarified cell culture supernatant. A VLP yield of 50% (measured by NTA in fluorescence mode) was obtained with a 17‐fold concentration factor regarding the loading material. Assuming a dose of 10^9^ particles, more than 44 doses can be captured per mL column.

This strategy streamlines VLP DSP process development and optimization and minimizes the need for several time‐consuming and laborious analytical techniques during severe viral outbreaks.

## CONFLICT OF INTEREST

The authors have declared no conflict of interest.
